# Viscous dissipation effect on unsteady magneto-convective heat-mass transport passing in a vertical porous plate with thermal radiation

**DOI:** 10.1016/j.heliyon.2023.e14207

**Published:** 2023-03-01

**Authors:** Md Hasanuzzaman, Sathi Akter, Shanta Sharin, Md Mosharof Hossain, Akio Miyara, Md Amzad Hossain

**Affiliations:** a Department of Mathematics, Khulna University of Engineering & Technology, Khulna, 9203, Bangladesh; b Department of Mathematics, Bangladesh University of Engineering and Technology, Dhaka, 1000, Bangladesh; c Department of Mechanical Engineering, Saga University, Saga-shi, 840-8502, Japan; d Department of Electrical and Electronic Engineering, Jashore University of Science and Technology, Jashore, 7408, Bangladesh

**Keywords:** MHD, Viscous dissipation, Thermal radiation, Heat and mass transfer, Permeability

## Abstract

The effects of radiative and viscous dissipation on the transfer of unsteady magnetic-conductive heat-mass across a vertically porous sheet is studied in this article. The non-dimensional ODEs are solved by applying the Finite Difference Method (FDM) through the MATLAB software numerically. The fluid temperature and velocity enhance for uplifting values of the Eckert number. Enhancing values of the transpiration parameter the velocity, concentration, and temperature distributions reduce. The local skin friction enhances about 9%, and 18% due to increase the Eckert number (0.5–3.0) and Dufour number (0.5–4.0), respectively and reduces 17%, 38%, and 31% due to increase Prandtl number (0.71–7.0), magnetic force parameter (0.5–3.0), and suction parameter (0.5–3.0), respectively. Enhancing values of the Eckert number (0.5–3.0) reduces the heat transfer rate by 40%. The increasing value of the Prandtl number (0.71–7.0) and the suction parameter (0.5–3.0) increases the heat transfer rate by 27% and 92%, respectively. With an increase in the values of the Schmidt number (0.22–0.67), the mass transfer rate increased by approximately 94%. At last, the numerical results of this paper has compared with the previously published paper. We noticed that the comparison has an excellent acceptance.

## 1 Introduction

The viscosity needs the highest consideration in the study of fluid flow for all the fluid properties. In the 20th century [1], introduced a new area of fluid dynamics by considering viscosity and thus theoretical hydrodynamics and unifying hydraulics. Many researchers carried on MHD (magnetohydrodynamics) flow of the boundary layer problem or a radiating gas inside a vertical pathway. The impact of this type of viscous dissipation term on an unsteady condition was often ignored. The influence of this heat dissipation function cannot be neglected from a practical point of view because of its momentous in several flow issues. It is the bearing lubricant that provides the source of temperature rise and geodynamic heating. For much lower velocity methods the impact of viscous dissipation in the temperature profile is comparatively small. The influence of viscous dissipation cannot be neglected in the manner concerning a dynamic temperature which is analogous to the attributed difference in heat transfer temperature. The boundary layer theory is utilized to analyze the viscous dissipation effect for both incompressible and compressible flows. The impact of variable fluid properties and dissipation function on time-dependent hydromagnetic radiating gas flow inside a vertical sheet has been identified by Ref. [2]. [3] analyzed the influence of constant suction on the time-dependent natural convection flow of an elastics viscous fluid past an infinite permeable sheet. The impacts of chemical reactions and MHD on electrically conductive, compressible, and viscous liquid volatile mass and heat transfer flow on a vertically permeable plate in a slip-flow area have been studied by Ref. [4]. [5] have included the influence of viscous removal on the free-flowing flow of an insoluble fluid flow on an infinitely vertical sheet. The impact of wall conduction and viscous dissipation on combined convection flow of heat generating or absorbing fluid has been investigated by Ref. [6]. The Soret number effect on the hydromagnetic natural circulation and mass transfer flow of electrically conductive non-Newtonian liquids in a vertically moving permeable sheet has been explored by Ref. [7].

The radiative heat transfer effect may not be omitted. Also, the impacts of the radiative on hydromagnetic mass and heat transfer flow will be more important in industrial regions. Many systems in science and engineering can happen at high temperatures. The idea of radiative heat transfer will be very significant for the relevant instrument model. The quality of the resultant product is largely dependent on heat-controlling factors. The concept of radiative heat transfer in the process may lead to desired products with sought-after properties. Unstable magnetic-conductive heat-filled transport over a permeable space with radiative effect has been investigated by Ref. [8]. The two types of chemical effects like homogeneous as well as heterogeneous in various practical applications. Mass transfer in homogeneous and heterogeneous reactions takes place through diffusive action. These reactions involve molecular diffusion of species. A heterogeneous reaction takes place within a phase boundary or confined region. On the other hand, a homogeneous reaction resembles a heat generation. It happens uniformly all over a given level. The rate of first-order reaction in the chemical reaction is directly proportionate to the concentration field. Dispersed species may be adsorbed or produced caused by a variety of chemical reactions with ambient fluids effects. It can be greatly affected by the quality and characteristics of the finished product. The effect of chemical reactions and magnetic fields on the flow of unstable natural circulation fluid from a vertically perforated sheet under thermal diffusion impact were studied by Ref. [9]. Further [10], observed a transverse magnetic field effect on a time-dependent free convection flow over a vertical plate. Also, they investigated the numerical results for the effects of heat sources and thermal diffusion on their system, along with radiative and diffusion-thermo effects [11]. analyzed the influences of chemical reaction and radiative on the 3D MHD viscous and fluid flow. They also added the Soret and Dufour impacts in their system. The effects of radiative and magnetic fields on a transient free circulating nanofluid that flows toward a vertical plate have been discussed by Ref. [12]. The blended effects of Joule heating, viscous dissipation, and radiative on the time-independent 2D electrical hydromagnetic boundary layer nano-fluids flow past a permeable linear stretching plate studied by Ref. [13]. They also solved the coupled ODEs by exerting the Keller box procedure. So the knowledge of radiation plays a significant role. Hence, the radiation's effect may not be ignored. Also, many relevant tools for system design and advanced energy conversions occur at high temperatures which is of major importance in the numerous methods of radiative in engineering. A formula is applied to interpret the radiant heat flux in the energy equation which is called the Roseland approximation [14]. performed a comparative study on hybrid, and conventional nanofluids adjust by Cu and Al_2_O_3_ including water as a base solvent. Further, because of their widespread application, heat transport in colloidal suspensions under different conditions has become an influential research aspect has been discussed by Ref. [15]. [16] studied an analogous research of entropy optimization on Newtonian and non-Newtonian fluid with heat and mass transfer of continuous MHD slip past a porous medium upon a melting stretching surface. The effect of heat source/sink and variable viscosity on MHD flow over a stretching surface embedded in a permeable medium has been studied by Ref. [17]. They used the Lie similarity analysis in their simulation [18]. examined the effect of modified Fourier's law in the non-linear combined convective third-grade liquid flow. The effect of variable thermal conductivity upon an impenetrable surface stretching in a nonlinear manner has been reported by Ref. [19]. The flow formulation is created by considering rheological statements of second-grade liquid and stagnation point.

The fluid flow is governed by concentration differences produced by simultaneous material formations, density gradients, and temperature gradients in combination with mass and heat transfer mechanisms. The diffusion-thermo's (Dufour) influence is the mass difference that generates the heat flux. The thermal-diffusion's (Soret) influence is the heat gradient that generates the mass flux. The Soret effect has been used to separate isotopes between medium molecular weights and gases including very light molecular weights in mixtures. The Dufour and Soret effects have encountered a lot of practical applications, like in the fields of geosciences and chemical engineering. The radiative and transpiration effects on MHD mixed convection and mass transfer fluid flow embedded in a non-Darcian saturated permeable medium have been studied by Ref. [20]. [21] discussed the Soret effect on hydromagnetic time-independent free heat and mass transport embedded in a permeable medium including the Dufour effect upon a vertical permeable sheet [22]. explained the influences of Dufour and Soret on the time-dependent free-flowing and mass transfer flow in infinitely vertical perforated flat sheets through a perforated medium. The magnetic field effect is transversely applied in their simulations. The effects hall currents, Dufour, Soret, and radiative on MHD flow by combined convective heat flux on a vertical surface in porous media were investigated by Ref. [23]. [24] discussed the entropy optimization and heat transfer effects on the magnet-nanomaterial flow of non-Newtonian fluids towards a curved moving surface. The effect of second-order velocity slip on nonlinear micropolar ferrofluid flow subject to the moving plate including heat source or sink, ohmic heating, and dissipation has been investigated by Ref. [25]. [26] discussed the time-dependent natural convective heat transfer flow around the boundary layer around a vertical slender body in presence of the transpiration [27]. explained the effects of Dufour and thermal scattering on unstable free magnet-conductive heat-mass transport across infinitely vertical perforated sheets. They solved the ODEs by utilizing the shooting technique through the MATLAB software. In this paper, we extended [27] by considering the additional term viscous dissipation. We observed the effect of the viscous dissipation on the velocity, temperature, and concentration profiles. Also, we are interested to observe the impact of this viscous dissipation term on heat transfer rate, skin friction coefficient, and mass transfer rate throughout the boundary of the permeable plate.

The prime purpose of this research is to take into account the above issues upon a vertically perforated plate considering the effects of viscous dissipation. The comparison of our numerical results with published paper is the main novelty of this paper. We have also improved this paper further by assuming the radiative and viscous dissipation through the Finite Difference Method (FDM) which is not been analyzed yet. This current work is to extend the works of [22] by considering the radiative and viscous dissipation. Non-dimensional numbers/parameters have been calculated for a wide range such as Eckert number, heat radiation, Schmidt number, magnetic force parameter, and Prandtl number explained graphically. Furthermore, mass, and heat transfer rates and the local friction coefficient are narrated with tabular structures.

## 2 Model and mathematical formulations

**Fig. 1:**
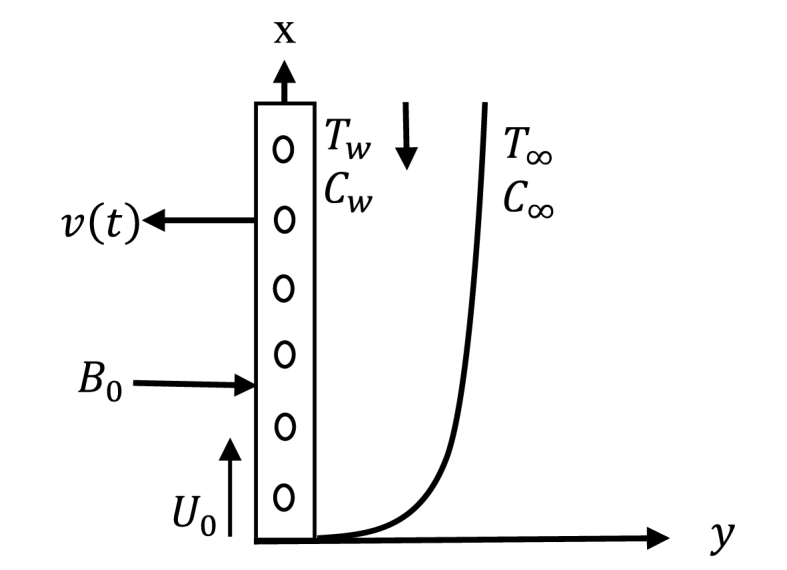
Physical model and coordinates system.

The unsteady 2D hydro-magnetic flow of an incompressible viscous and electrically conducting fluid along a permeable vertical flat sheet joined in a permeable medium is considered. The fluid flow direction is on the x-axis. The free stream velocity is parallel to this axis which is vertical. The vertical porous sheet is perpendicular to the y-axis. A magnetic field with uniform strength B is transversely applied to the flow direction. The permeable sheet begins passing impulsively in its self-plane with a velocity $U_{0}$ for t>0. The fluid temperature at the sheet is increased to $T_{w}$. The fluid concentration at the sheet is increased to $C_{w}$. The physical model and coordinate systems are plotted on Fig. 1 [26]. The fluid is considered to have certain properties except for the effects of concentration variation with temperature and concentration. This effects are assumed only in terms of physical forces. The velocity components are the function of y and t.

We assume the applied uniform magnetic field is remarkably small. The magnetic Reynolds number has been compared to one of the studies [28]. Then the magnetic force lines are $B=\left(0,B_{0},0\right)$. The density of current is $J=\left(J_{x},J_{y},J_{z}\right)$. The charge continuity equation is given by ∇.J=0. The solution of this equation is $J_{y}=$ constant. The *y*-axis is considered along only the propagation direction. There is no change in the direction of this propagation along the y-axis. For this reason $\frac{\partial J_{y}}{\partial y}=0$. Since the plate is electrically non-conductive then this constant of integration is zero, so $J_{y}=0$ at the plate.

Considering that the boundary-layer as well as Boussinesq approximation, the equations of governing fluid flow are given as [26]:

(1) $$\frac{\partial v}{\partial y}=0$$

(2) $$\frac{\partial u}{\partial t}+v\frac{\partial u}{\partial y}=\upsilon \frac{\partial^{2}u}{\partial y^{2}}+g\beta \left(T-T_{\infty }\right)+g\beta^{*}\left(C-C_{\infty }\right)-\frac{\sigma^{\prime }B_{0}^{2}u}{\rho }-\frac{\upsilon }{K}u-\frac{b}{K}u^{2}$$

(3) $$\frac{\partial T}{\partial t}+v\frac{\partial T}{\partial y}=\frac{k}{\rho C_{p}}\frac{\partial^{2}T}{\partial y^{2}}+\frac{D_{m}k_{T}}{C_{s}C_{p}}\frac{\partial^{2}C}{\partial y^{2}}-\frac{1}{\rho C_{p}}\frac{\partial q_{r}}{\partial y}-\frac{\nu }{\rho C_{p}}{\left(\frac{\partial u}{\partial y}\right)}^{2}$$

(4) $$\frac{\partial C}{\partial t}+v\frac{\partial C}{\partial y}=D_{m}\frac{\partial^{2}C}{\partial y^{2}}+\frac{D_{m}k_{T}}{T_{m}}\frac{\partial^{2}T}{\partial y^{2}}$$

Boundary conditions are given as:

(5) $$t>0,u=U_{0}\left(t\right),T=T_{w},v=v\left(t\right),C=C_{w}\;\text{at}\;y=0$$

(6) $$t>0,u=0,T\to T_{\infty },v=0,C\to C_{\infty }\;\text{at}\;y\to \infty$$

where T = fluid temperature ,u = velocity components in the x−axis, v = velocity components in the y−axis, C = fluid concentration, β = volumetric expansion coefficient including temperature, $T_{w}$ = wall temperature, $C_{w}$ = wall concentration, ρ = fluid density, $C_{\infty }$ = fluid concentration in the free stream, k = plate thermal conductivity, $T_{\infty }$ = fluid temperature in the free stream, $C_{s}$ = concentration susceptibility, $C_{p}$ = specific heat at constant pressure, the $k_{T}$ = thermal diffusion ratio, υ = fluid kinematic viscosity, g = acceleration owning to gravity, $D_{m}$ = mass diffusivity coefficient, $T_{m}=$ mean temperature of the fluid, and $\beta^{*}$ = expansion of volumetric coefficient with concentration, $q_{r}$ = component of radiative heat flux.

Assuming a similarity parameter σ which is given by

(7) σ=σ(t)

where σ is the unsteady length scale. The solution of continuity equation (1) is assumed in terms of this unsteady length scale. This solution is given by the following relation:

(8) $$v=-v_{0}\frac{\upsilon }{\sigma }$$

Here $v_{0}<0$ displays blowing and $v_{0}>0$ displays suction, where $v_{0}$ is the non-dimensional normal velocity at the sheet.

The radiative heat flux $\left(q_{r}\right)$ [29] is given as:

$$q_{r}=-\frac{4\sigma^{*}}{3K^{*}}\frac{\partial T^{4}}{\partial y}$$

where $K^{*}$ is the coefficient of mean absorption and $\sigma^{*}$ is the constant of Stefan-Boltzmann.

We consider from Ref. [30] that the difference between the liquid temperature and the free flow temperature is quite small.

Now, $T^{4}$ is being expanded in a Taylor series about $T_{0}$. We ignored the higher-order terms yield:

$$T^{4}≅4T_{0}^{3}T-3T_{0}^{4}$$

We used the following similarity transformations [16]:

(9) $$\eta =\frac{y}{\sigma },\theta \left(\eta \right)=\frac{T-T_{\infty }}{T_{w}-T_{\infty }},f\left(\eta \right)=\frac{u}{U_{0}},\varphi \left(\eta \right)=\frac{C-C_{\infty }}{C_{w}-C_{\infty }}$$

The governing equations (1), (2), (3), (4) are transformed into the non-dimensional coupled ODEs by using the above equations (7), (8), (9)

(10) $$f^{\prime \prime }\left(\eta \right)+2\xi f^{\prime }\left(\eta \right)+Gr\theta \left(\eta \right)+Gm\varphi \left(\eta \right)-Mf\left(\eta \right)=0$$

(11) $$\theta^{\prime \prime }\left(\eta \right)+\frac{\mathrm{Pr}}{1+R}\left[2\xi \theta^{\prime }\left(\eta \right)+Ec{\left\{f^{\prime }\left(\eta \right)\right\}}^{2}+Df\varphi^{\prime \prime }\left(\eta \right)\right]=0$$

(12) $$\varphi^{\prime \prime }\left(\eta \right)+Sc\left[2\xi \varphi^{\prime }\left(\eta \right)+Sr\theta^{\prime \prime }\left(\eta \right)+Kr\varphi \left(\eta \right)\right]=0$$

The corresponding transformed boundary conditions are given by

(13) θ=1,f=1,φ=1atη=0

(14) θ=0,f=0,φ=0atη→∞

where $Gr=\frac{g\beta \left(T_{w}-T_{\infty }\right)\sigma^{2}}{U_{0}\upsilon }$ = local Grashof number, $M=\frac{\sigma^{\prime }B_{0}^{2}\sigma^{2}}{\rho \upsilon }=$ magnetic force parameter, $\mathrm{Pr}=\frac{\rho \upsilon C_{p}}{k}=$ Prandtl number, $Df=\frac{D_{m}k_{T}\left(C_{w}-C_{\infty }\right)}{C_{s}C_{p}\upsilon \left(T_{w}-T_{\infty }\right)}=$ Dufour number, $Gm=\frac{g\beta^{*}\left(C_{w}-C_{\infty }\right)\sigma^{2}}{U_{0}\upsilon }=$ modified local Grashof number, $Sr=\frac{D_{m}k_{T}\left(T_{w}-T_{\infty }\right)}{\upsilon T_{m}\left(C_{w}-C_{\infty }\right)}=$ Soret number, $Sc=\frac{\upsilon }{D_{m}}=$ Schmidt number, $Ec=\frac{U_{0}^{2}}{\left(T_{w}-T_{\infty }\right)C_{p}}=$ Eckert number and $\xi =\eta +\frac{v_{0}}{2}$.

The Significant physical quantities need to be noted, for example, local skin friction $\left(f^{\prime }\left(0\right)\right)$, Nusselt number $\left(N_{u}\right)$ and Sherwood number $\left(S_{h}\right)$ given by the following relationships:

(15) $$\tau \propto f^{\prime }\left(0\right),N_{u}\propto -\theta^{\prime }\left(0\right),S_{h}\propto -\varphi^{\prime }\left(0\right)$$

## 3 Numerical solution

The principal aim of this study is to apply the FDM (Finite Difference Methods) for solving the couple ODEs (10)–(12) corresponding boundary conditions (13)–(14). Such methods are tested for efficiency and accuracy in solving various problems [31,32]. The solution domain location is discretized in FDMs (Finite Difference Methods). Let the grid size is Δη=h>0 in η-direction and $\Delta \eta =\frac{1}{N}$, with $\eta_{i}=ih$ for *i* = 0,1, …,*N*. Define $f_{i}=f\left(\eta_{i}\right)$, $\theta_{i}=\theta \left(\eta_{i}\right)$ and $\varphi_{i}=\varphi \left(\eta_{i}\right)$.

Let the numerical values of f,θandφ are $F_{i}$, $\Theta_{i}$ and $\Phi_{i}$ at the $i^{th}$ node, respectively. We take:

(16) $${f^{\prime }|}_{i}=\frac{f_{i+1}-f_{i-1}}{2h},{\theta^{\prime }|}_{i}=\frac{\theta_{i+1}-\theta_{i-1}}{2h},{\varphi^{\prime }|}_{i}=\frac{\varphi_{i+1}-\varphi_{i-1}}{2h}$$

(17) $${f^{\prime \prime }|}_{i}=\frac{f_{i+1}-2f_{i}+f_{i-1}}{h^{2}},{\theta^{\prime \prime }|}_{i}=\frac{\theta_{i+1}-2\theta_{i}+\theta_{i-1}}{h^{2}},{\varphi^{\prime \prime }|}_{i}=\frac{\varphi_{i+1}-2\varphi_{i}+\varphi_{i-1}}{h^{2}}$$

the system of ODES (10), (11), and (12) is separated in space by applying the FDM which is the principal step. We get (10), (11), and (12) from (16), (17) into and delete the truncation errors. Then for (*i* = *0, 1, …, N*), the final algebraic equations may take the form:

(18) $$F_{i+1}-2F_{i}+F_{i-1}+h\xi \left(F_{i+1}-F_{i-1}\right)+h^{2}\left(Gr\Theta_{i}+Gm\Phi_{i}-MF_{i}\right)=0$$

(19) $$\left(1+R\right)\left(\Theta_{i+1}-2\Theta_{i}+\Theta_{i-1}\right)+\mathrm{Pr}\left[\xi h\left(\Theta_{i+1}-\Theta_{i-1}\right)+Ec{\left\{\left(F_{i+1}-F_{i-1}\right)\right\}}^{2}+Df\left(\Phi_{i+1}-2\Phi_{i}+\Phi_{i-1}\right)\right]=0$$

(20) $$\Phi_{i+1}-2\Phi_{i}+\Phi_{i-1}+Sc\left[\xi h\left(\Phi_{i+1}-\Phi_{i-1}\right)+Sr\left(\Theta_{i+1}-2\Theta_{i}+\Theta_{i-1}\right)+Krh^{2}\Phi_{i}\right]=0$$

Also, the boundary conditions are

(21) $$F_{0}=1,\Phi_{0}=1,\Theta_{0}=1,F_{N}=0,\Phi_{N}=0,\Theta_{N}=0$$

The system of equations (18), (19), (20) is a nonlinear system of algebraic equations in $F_{i},\Theta_{i},\text{and}\;\Phi_{i}$. We have solved these nonlinear system of algebraic equations by including the Newton iteration method through the MATLAB software.

## 4 Results and discussions

**Fig. 2:**
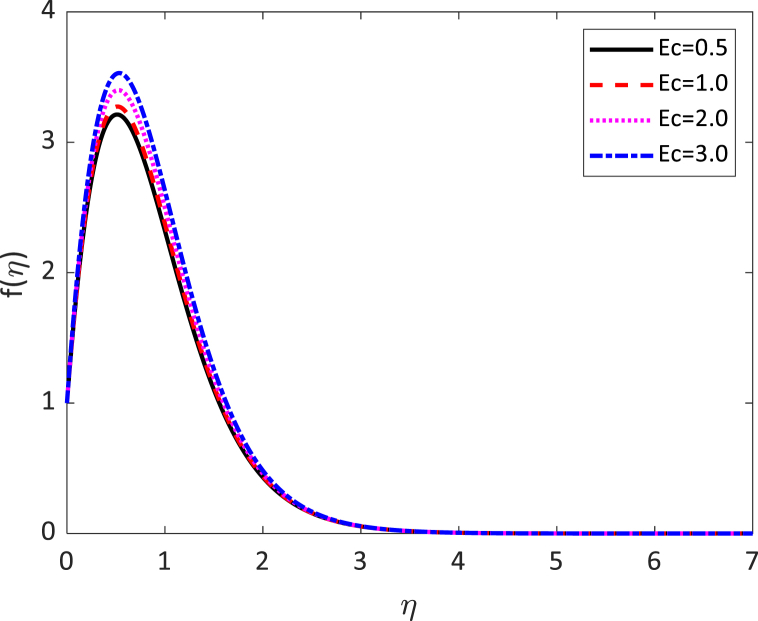
Velocity profile for *E*_*c*_.

**Fig. 3:**
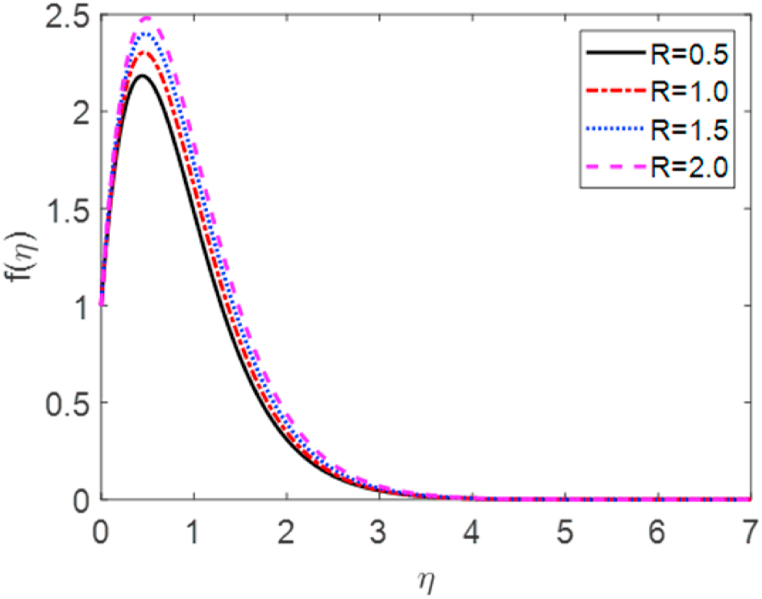
Velocity profile for *E*_*c*_.

**Fig. 4:**
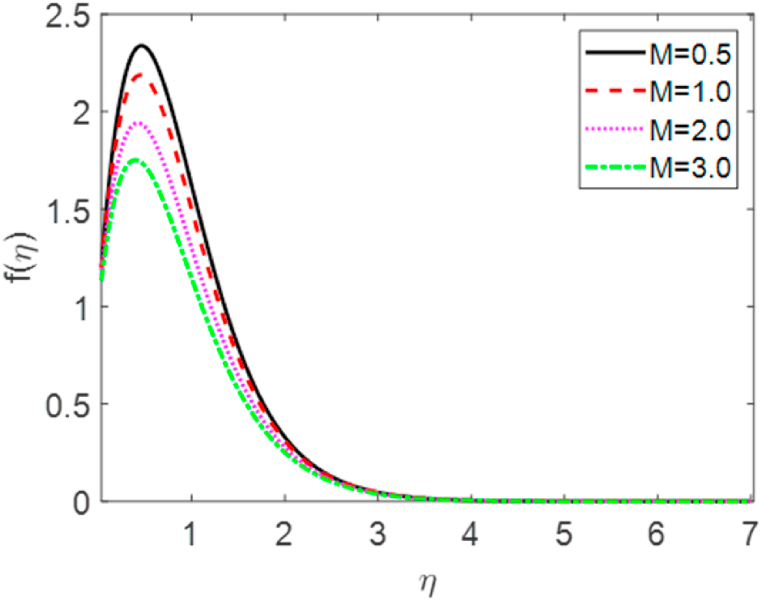
Velocity profile for M.

**Fig. 5:**
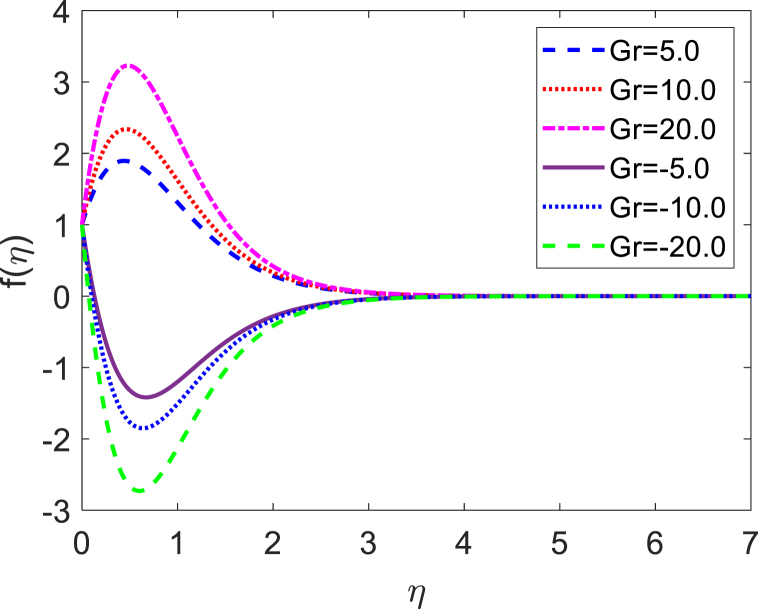
Velocity profile for *Gr*.

**Fig. 6:**
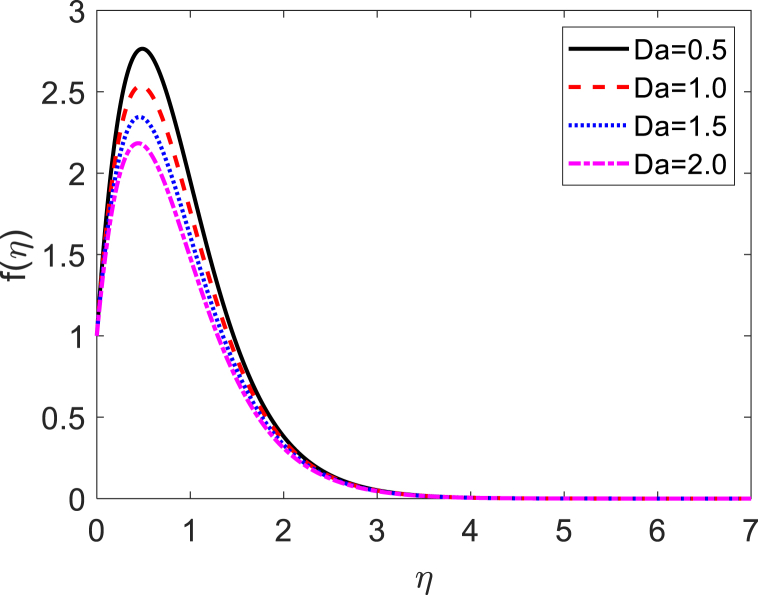
Velocity profile for *Da*.

**Fig. 7:**
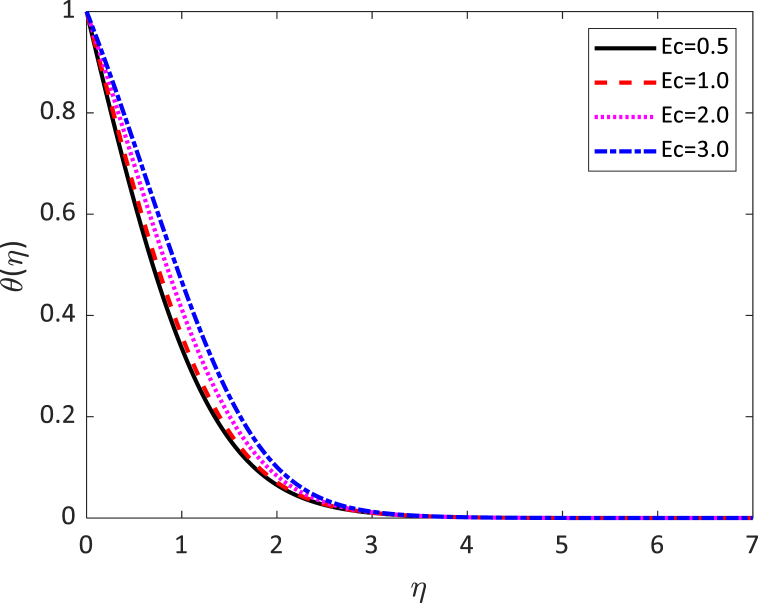
Temperature profile for *E*_*c*_.

**Fig. 8:**
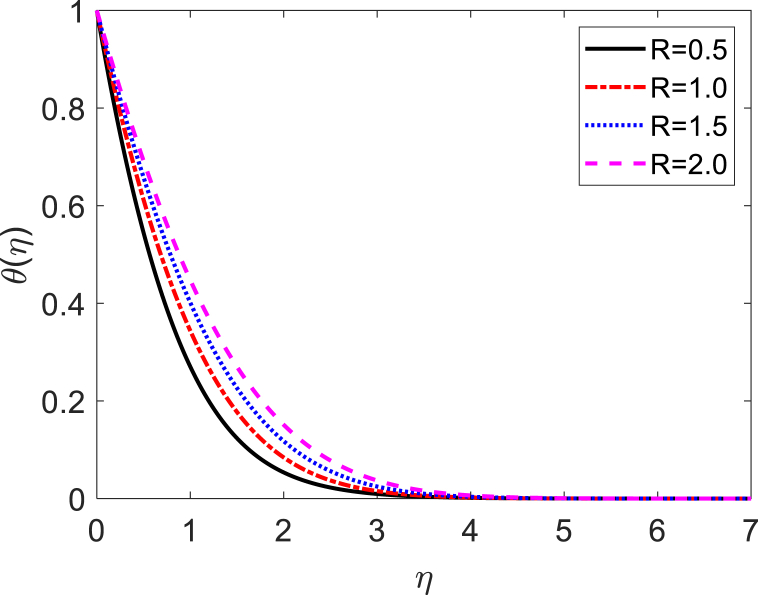
Temperature profile for *R*.

**Fig. 9:**
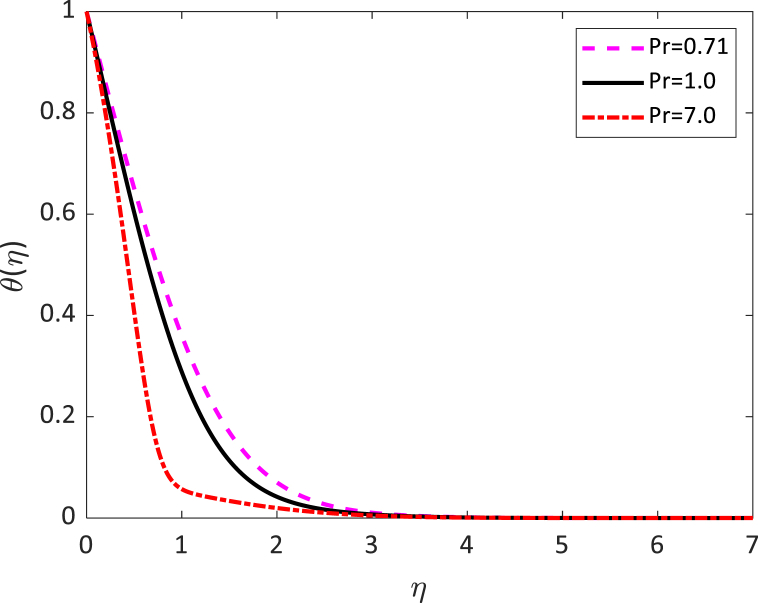
Temperature profile for *Pr*.

**Fig. 10:**
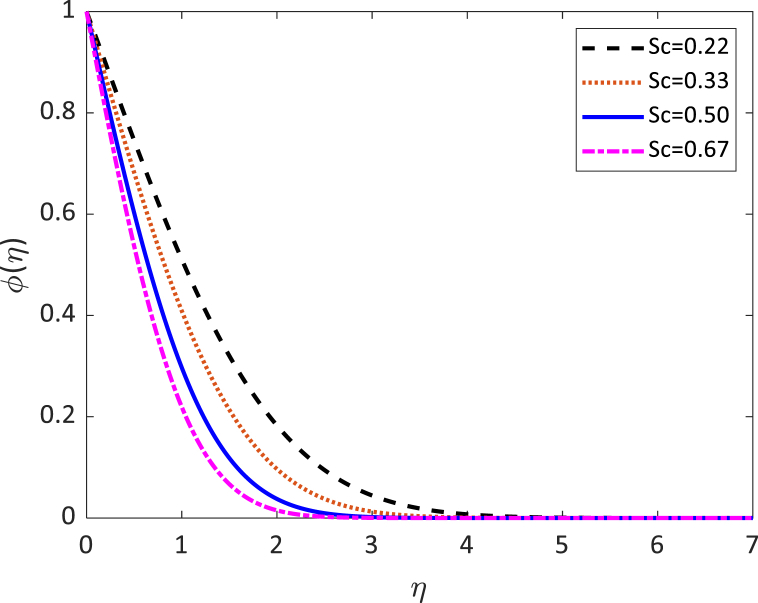
Concentration profile for (*S*_*c*_).

In this numerical analysis, the viscous dissipation effect on time-dependent magneto-convective mass and heat transport through a vertical permeable sheet in presence of the radiative is discussed. The initial valued problems with the set ODEs (10)–(12) are numerically resolved using the “MATLAB ODE45″ software using the limiting difference method (FDM) associated with the boundary conditions (13)–(14). The Eckert number (Ec), the Grashof number (Gr) the magnetic parameter (M), the radiation parameter (R), the Darcy number (Da) the Prandtl number (Pr), and the Schmidt number (Sc) effects on temperature, concentration, and velocity profiles are exhibited in Fig. 2, Fig. 3, Fig. 4, Fig. 5, Fig. 6, Fig. 7, Fig. 8, Fig. 9, Fig. 10. The values 0.71, 1.0, and 7.0 are assumed for Pr (0.71 for air at 20°, and 1.0, 7.0 for water at 17° and 20°, respectively). Also, the values 0.75, 0.60, and 0.22are also supposed for Sc (0.75 for Oxygen, 0.60 for vapor water, and 0.22 for Hydrogen). The rest of the number or parameter values are taken as constant. In the absence Eckert number as Ec=0 the values of the Sherwood number, the skin friction, and the Nusselt number are compared with the numerical results of Alam et *al*. [16]**.** It is seen that they are in good contract which is displayed in Table 9 and Table 10, respectively. The various non-dimensional parameters/numbers values are chosen as $v_{0}=0.5,G_{r}=10.0,G_{m}=10.0,M=0.5,\mathrm{Pr}=0.71,Da=0.5,R=1.0,K_{r}=0.5,Sc=0.22,Df=0.5\;\text{and}\;Sr=1.0$.

### 4.1 Velocity profiles for several values of non-dimensional numbers/parameters

Velocity distribution for several values of the Eckert number (Ec) is shown in Fig. 2, Fig. 6. The effect of the Eckert number is to create more skin friction coefficients in the border layer area and to improve the fluid velocity as shown in Fig. 2. Larger viscous dissipative heat enhances the temperature profile. The nature of radiative parameter (R) on the velocity profile is exhibited in Fig. 3. The radiative parameter is defined $R=\frac{16\sigma^{*}T_{W}^{2}}{3k^{*}k}$ and shapes in the improved thermal diffusion term in Eqn. (11) i.e. $\frac{1}{\mathrm{Pr}}\left(1+R\right)\theta^{\prime \prime }\left(\eta \right)$. The comparative contribution of radiative with thermal conductive heat transfer is defined by the radiative parameters. When R>1, then the radiative dominates over thermal conduction, on the other hand the thermal conduction dominates for R<1. The thermal conduction and radiative contributions both are equal when R=1. We considered only the case R > 1 for our current study. Fig. 3 shows that a powerful acceleration of linear velocity as *R* increases. Strengthening the flow results in increased thermal expansion and then decreasing velocity expansion. This momentum leads to a reduction in the boundary layer thickness. Fig. 4 illustrates the velocity profile for several values of the magnetic force parameter (M). It is seen from Fig. 4 that the velocity of the fluid decays due to the rising value of M. Increasing the value of M results in the formation of a resistive force. This resistive type of force is said to be Lorentz force (drag force). This Lorentz force interrupts the fluid velocity which decays its fluid motion. So, the fluid velocity decreases for rising values of M. For improving values of M the fluid velocity decreases which is a physical phenomenon. Fig. 5 illustrates the velocity profile for the individual values of the local Grashof number (*Gr*). It is found from Fig. 5 shows that the rising values of the local gravitational number enhances the value of the wave velocity due to the increase in the wave force. The velocity profile formed a symmetrical form for negative and positive values of the local Grashof number. We observed that when the mode of heating is increased, the velocity also increases but a converse impact is observed for the case of cooling. Consequently, symmetric figures are obtained. It is also well established that the improvement of buoyancy parameters improves the fluid flow. The impact of Darcy number (*Da*) on velocity profile is revealed in Fig. 6. Fig. 6 shows that the velocity of the liquid exacerbates with increasing the value of Da. For huge values of the Darcy number, the permeability of the medium decays, so the fluid flows slowly.

### 4.2 Temperature profiles for separate values of non-dimensional parameters or numbers

Fig. 7 exhibits the impact of several values of the Eckert number on the temperature distribution. We noticed from Fig. 7 that the fluid temperature increases due to the increase in the value of the Eckert number. It is compatible with energy storage in fluid areas as a result of removal because of elastic and viscosity deformation. Fig. 8 exhibits the influence of radiative parameter (R) on the temperature distribution. From Fig. 8 it can be observed that the surface temperature gradient decreases to increase the value of radiative parameters. The heat transfer rate reduces due to the improving values of R on the surface. The radiative parameter is liable for thickening the thermal boundary. The fluid flow gives up the heat energy from the flow zone and as a result, the system cools down. Fig. 9 shows the influence of the Prandtl number (Pr) on the temperature distribution. This is because the Rosseland approximation increases the temperature. The thermal conductivity is inversely proportional to the Prandtl number (Pr). As the value of thermal conductivity decreases, the Prandtl number increases. From Fig. 8, it is seen that the temperature profile diminishes by reducing values of the thermal conductivity. In practically, a higher Prandtl number has relatively a lower thermal conductivity, which reduction in the thermal conductivity, and therefore the temperature decreases. For this reason, the heat transfer rate enhances as the magnitude of *Pr* increases. Hence, the fluid temperature diminishes.

### 4.3 Concentration profile for separate values of schmidt number

Fig. 10 presents the effect of the separate values of the Schmidt number (Sc) on the concentration profile. The Schmidt number is inversely proportionate to the molecular (species) diffusivity. The momentum level and density (species) will have the same thickness and diffusivity rate for Sc=1. When Sc>1 then the rate of reproduction of the momentum overcomes the rate of reproduction of the species. When Sc<1 then the opposite behavior happened. The concentration profile in Fig. 10 shows the decrease in concentration for the improving values of the Schmidt number. The reduction associated with mass diffusivity leads to a smaller force mass transfer which reduces the density level. So, the thickness of the concentration boundary layer reduces. Therefore, the mass transfer applies interplay with the field of velocity and the distribution of species in the matter may be dominated by the Schmidt number.

## 5 Local skin friction coefficient, heat transfer rate and mass transfer rate

The authors are interested in discussing not only the field of concentration, velocity as well as temperature but also the values of the mass transfer rate, local skin friction coefficient as well as heat transfer rate. Table 1, Table 2, Table 3, Table 4, Table 5, Table 6, Table 7, Table 8 represents the influence of different values of dimensionless numbers or parameters on the mass transfer rate, local skin friction coefficient, and heat transfer rate.

**Table 1:** Local skin friction coefficient, heat, and mass transfer rates for several values of the Eckert number (*E*_*c*_).

Ec	$f^{\prime }\left(0\right)$	$-\theta^{\prime }\left(0\right)$	$-\varphi^{\prime }\left(0\right)$
0.5	9.73434042713189	0.789503210657104	0.507185477215647
1.0	9.90714797340817	0.708822984077260	0.507185477215647
2.0	10.2535310321875	0.578457484667413	0.507185477215647
3.0	10.5973676674043	0.479694060727014	0.507185477215647

**Table 2:** Local skin friction coefficient, heat, and mass transfer rates for several values of the Magnetic force parameter (*M*).

M	$f^{\prime }\left(0\right)$	$-\theta^{\prime }\left(0\right)$	$-\varphi^{\prime }\left(0\right)$
0.5	9.90714797340817	0.708822984077260	0.507185477215647
1.0	8.92455399469696	0.708822984077260	0.507185477215647
2.0	7.38000471889979	0.708822984077260	0.507185477215647
3.0	6.20754853985141	0.708822984077260	0.507185477215647

**Table 3:** Local skin friction coefficient, heat, and mass transfer rates for several values of the suction (*v*_0_).

$v_{0}$	$f^{\prime }\left(0\right)$	$-\theta^{\prime }\left(0\right)$	$-\varphi^{\prime }\left(0\right)$
0.5	9.90714797340817	0.708822984077260	0.507185477215647
1.0	9.53464934445465	0.827498607955273	0.586804301457662
2.0	8.37591551753257	1.08344344690441	0.755419696776116
3.0	6.88883709533525	1.35872949128436	0.934224084312002

**Table 4:** Local skin friction coefficient, heat, and mass transfer rates for several values of the Grashof number (*Gr*).

Gr	Gm	$f^{\prime }\left(0\right)$	$-\theta^{\prime }\left(0\right)$	$-\varphi^{\prime }\left(0\right)$
.0	10.0	7.29708880447365	0.708822984077260	0.507185477215647
10.0	10.0	9.90714797340817	0.708822984077260	0.507185477215647
30.0	10.0	20.3473846489773	0.708822984077260	0.507185477215647
−5.0	−10.0	−10.5698484622934	0.708822984077260	0.507185477215647
−10.0	−10.0	−13.1799076313860	0.708822984077260	0.507185477215647
−30.0	−10.0	−23.6201443070609	0.708822984077260	0.507185477215647

**Table 5:** Local skin friction coefficient, heat and mass transfer rates for several values of the modified Grashof number (*G*_*m*_).

Gm	Gr	$f^{\prime }\left(0\right)$	$-\theta^{\prime }\left(0\right)$	$-\varphi^{\prime }\left(0\right)$
5.0	10.0	6.74544323998706	0.708822984077260	0.507185477215647
10.0	10.0	9.90714797340817	0.708822984077260	0.507185477215647
30.0	10.0	22.5539669029845	0.708822984077260	0.507185477215647
−5.0	−10.0	−10.0182028988166	0.708822984077260	0.507185477215647
−10.0	−10.0	−13.1799076313860	0.708822984077260	0.507185477215647
−30.0	−10.0	−25.8267265610733	0.708822984077260	0.507185477215647

**Table 6:** Local skin friction coefficient, heat, and mass transfer rates for several values of the Prandtl number (*Pr*).

Pr	$f^{\prime }\left(0\right)$	$-\theta^{\prime }\left(0\right)$	$-\varphi^{\prime }\left(0\right)$
0.71	9.90714797340817	0.708822984077260	0.507185477215647
1.0	9.46549480161802	0.797556028694775	0.507185477215647
7.0	8.22538425462258	0.898517183405347	0.507185477215647

**Table 7:** Local skin friction coefficient, heat, and mass transfer rates for several values of the Dufour number (*Df*).

Df	$f^{\prime }\left(0\right)$	$-\theta^{\prime }\left(0\right)$	$-\varphi^{\prime }\left(0\right)$
0.5	9.90714797340817	0.708822984077260	0.507185477215647
2.0	10.6293856202926	0.600383715136317	0.507185477215647
3.0	11.1231012674706	0.523449570129626	0.507185477215647
4.0	11.6270721634308	0.442497835845966	0.507185477215647

**Table 8:** Local skin friction coefficient, heat, and mass transfer rates for several values of the Schmidt number (*Sc*).

Sc	$f^{\prime }\left(0\right)$	$-\theta^{\prime }\left(0\right)$	$-\varphi^{\prime }\left(0\right)$
0.22	9.73434042713189	0.708822984077260	0.507185477215694
0.33	9.00825617325291	0.708822984077260	0.642713920740657
0.5	8.31583873406485	0.708822984077260	0.824880790849183
0.67	7.86319821973989	0.708822984077260	0.988063441126695

The influence of several values of the dimensionless numbers or parameters like Eckert number (Ec), Magnetic force parameter (M), suction $\left(v_{0}\right)$, Grashof number (Gr), modified Grashof number (Gm), Prandtl number (Pr), Dufour number (Df) and Schmidt number (Sc) on the mass transfer rate, heat transfer rate as well as local skin friction coefficient have been interpreted in Table 1, Table 2, Table 3, Table 4, Table 5, Table 6, Table 7, Table 8 It is revealed from Table 1, Table 2, Table 3, Table 4, Table 5, Table 6, Table 7, Table 8, that the local skin friction coefficient enhances for improving values of the local Grashof number, Eckert number, local modified Grashof number, and Dufour number. Besides, the local skin friction coefficient decreases for growing values of Prandtl number, magnetic force number, Schmidt number, and suction parameter. The heat transfer rate improves for raising values of the suction parameter as well as Prandtl number. On the other hand, the reverse trends are shown for the Dufour number and Eckert number. Also, increase the mass transfer rate to improve the suction parameters as well as the value of the Schmidt number.

## 6 Comparison

We are interested in comparing our results with previously published papers [16]. Table 9, Table 10 illustrate the comparison of the local Sherwood number and the local Nusselt number. Some limited cases are evaluated with previously published results and we find good agreement.

**Table 9:** Comparison of local Sherwood number (Sh) for different values of So and Df when Ec=0 and *R* = 0.

		[16]	Present results	Persistence of error
So	Df	Sh	Sh	Sh
1.0	0.06	0.315615	0.31607639	0.046139
0.5	0.12	0.468128	0.46965549	0.152749
0.4	0.15	0.496002	0.49747809	0.147609
0.2	0.30	0.549515	0.54972859	0.021359
0.1	0.60	0.575236	0.57553013	0.029413

**Table 10:** Comparison of local Nusselt number (Nu) for different values of So and Df when Ec=0 and *R* = 0.

		[16]	Present results	Persistence of error
So	Df	Nu	Nu	Nu
1.0	0.06	1.652241	1.65241042	0.016942
0.5	0.12	1.541984	1.54403130	0.204730
0.4	0.15	1.517881	1.51849539	0.061439
0.2	0.30	1.450355	1.45560581	0.525081
0.1	0.60	1.364561	1.36587384	0.131284

## 7 Conclusions

The effects of condensate dissipation on the transfer of unstable magnetic-conductive heat-mass across a vertically permeable sheet with radiative effect has been analyzed. From the above numerical results, the following conclusions may be drawn.

• The velocity of the fluid improves for improving values of Eckert number (*Ec*), the radiative parameter (*R*) as well as the local Grashof number (*Gr*).
• The temperature of the fluid enhances for moving values of Eckert number (*Ec*) and the radiative parameter (*R*).
• The species decays for improving values of Sc.
• The local skin friction coefficient increases about 9%, 36%, and 18% for enhancing values of Eckert number (0.5–3.0), Grashof number (5.0–10.0), and Dufour number (0.5–4.0), respectively. Increasing values of magnetic force parameter (0.5–3.0), suction parameter (0.5–3.0), Prandtl number (0.71–7.0), and Schmidt number (0.22–0.67) the local skin friction reduces about 38%, 31%, 17%, and 20%, respectively.
• Increasing the values of the Eckert number (0.5–3.0), and Dufour number (0.5–4.0) resulted in a reduction of rate of the heat transfer by about 39% and 53%, respectively. The heat transfer rate for the increasing quality suction parameter (0.5–2.0) and the Prandtl number (0.71–7.0) increased by about 53%, and 27%, respectively.
• Owing to the increase in Schmidt number (0.22–0.67) and suction parameter (0.5–2.0) the mass transfer rate increased by about 94%, and 49% respectively.

The outcome results of this study may be helpful for geodynamic heating, bearings lubricant, drawing of plastic films, polymer sheet extrusion from a dye, geothermal energy extraction, geophysical flows, etc.

## Author contribution statement

Md. Hasanuzzaman, Ph. D: Conceived and designed the experiments; Analyzed and interpreted the data; Contributed reagents, materials, analysis tools or data; Wrote the paper.

Sathi Akter, M. *Sc*; Md Mosharrof Hossain, Mphil: Conceived and designed the experiments; Performed the experiments; Analyzed and interpreted the data; Contributed reagents, materials, analysis tools or data.

Shanta Sharin, M. *Sc*; Md Amzad Hossain, PhD: Conceived and designed the experiments; Performed the experiments; Analyzed and interpreted the data; Contributed reagents, materials, analysis tools or data; Wrote the paper.

Akio Miyara, PhD: Conceived and designed the experiments; Analyzed and interpreted the data; Contributed reagents, materials, analysis tools or data.

## Funding statement

This research did not receive any specific grant from funding agencies in the public, commercial, or not-for-profit sectors.

## Data availability statement

The data that has been used is confidential.

## Declaration of interest's statement

The authors declare no conflict of interest.
